# Parents’ Attitudes Toward and Experience of Non-Suicidal Self-Injury in Adolescents: A Qualitative Study

**DOI:** 10.3389/fpsyt.2020.00651

**Published:** 2020-07-15

**Authors:** Xi Fu, Jiaxin Yang, Xiaoli Liao, Jingjing Lin, Yao Peng, Yidong Shen, Jianjun Ou, Yamin Li, Runsen Chen

**Affiliations:** ^1^ National Clinical Research Center for Mental Disorders, Department of Psychiatry, and Hunan Medical Center for Mental Health, The Second Xiangya Hospital of Central South University, Changsha, China; ^2^ Clinical Nursing Teaching and Research Section, The Second Xiangya Hospital, Central South University, Changsha, China; ^3^ XiangYa Nursing School, Central South University, Changsha, China

**Keywords:** non-suicidal self-injury, adolescents, parents’ attitude, parents’ experience, qualitative

## Abstract

**Background:**

Non-suicidal self-injury (NSSI) is prevalent in adolescents and brings a series of serious consequences to their well-being. However, little is known about parents’ attitude toward NSSI in Chinese adolescents. The study aims to investigate the parents’ attitudes toward and perceptions of adolescents who have engaged in NSSI behaviors, and the impact of NSSI on their parents.

**Methods:**

Purposive sampling was used in the study. The biological parents of adolescents with NSSI were recruited from the psychiatric ward of a tertiary hospital in China. Semi-structured interviews were conducted which contained three aspects, that is the history of NSSI, the process of seeking or maintaining help and the impacts on the family. Each interview typically lasted 40–50 min. All of the interviews were audio-recorded. Their responses were analyzed by the thematic analysis.

**Results:**

Twenty participants completed the interview, consisting of 16 mothers and 4 fathers. Three themes and eight sub-themes were extracted: (1) the attitudes to children’s NSSI behaviors (ignorance, shame, and stereotype); (2) coping strategies of parents (the initial response to adolescents’ NSSI, and the way of help-seeking); (3) the impacts on family (altered parenting and communication styles, limited personal lives, and increased psychological pressure).

**Conclusion:**

The results showed that parents lack the knowledge about NSSI and its treatment and are suffering great emotional stress. It is recommended to expand the popularization of knowledge of NSSI in adolescents and more interventions adapted to China’s sociocultural climate are required for the well-being of parents and NSSI in adolescents.

## Introduction

Non-suicidal self-injury (NSSI), also called deliberate self-injury, refers to a self-injury behavior that is intentional, unsanctioned by society, and not intended for suicide (1). Common forms of NSSI include cutting, burning, scratching, pinching, biting, and poisoning, and such behavior is considered a way to relieve negative emotions, attract others’ attention, or exact revenge on or threaten someone (2, 3). NSSI can occur in any group, including adolescents, college students, and patients with mental illness. However, it is most common among adolescents, and the onset of NSSI is most often between 12 and 15 years old (4) The global prevalence of NSSI among adolescents is approximately 17% in nonclinical samples (5), with most cases occurring among females (6). In China, the prevalence of NSSI is higher than this, reaching 17.0%–29.2% in different community samples (7).

NSSI is known to be associated with a combination of factors (8). The most common co-occurrences with NSSI are depressive disorders and borderline personality disorder (BPD) (9). Nearly 50% of patients with NSSI were diagnosed with severe depressive disorder (10) and it was reported that 49%–90% of BPD patients have NSSI. Similarly, patients with anxiety disorders and eating disorders also have a high risk of NSSI (11). In addition, substance use is one of the antecedents leading to adolescent NSSI, and nearly 50% of adolescents with NSSI have a history of substance use (12). Moreover, individuals with NSSI are more likely to have specific temperamental profiles. Mitsui et al. reported that the lower the scores of self-directedness and cooperativeness in the temperament and character inventory (TCI) are, the higher the incidence of NSSI is (13). In addition to the internal factors, it is generally known that family provides a critical background in the process of the growth of adolescents. Previous studies have shown that the relationship with parents is one of the risk factors for adolescent NSSI behaviors (14), especially parenting style. A supportive and warm parenting style is associated with less NSSI (15), while adolescents under highly controlling parenting who do not perceive parental support are more likely to engaged in NSSI (16, 17).

Notably, parents’ attitudes toward NSSI have a significant impact on help-seeking. Prior work has demonstrated that the initial response of parents affects the timing of young people’s formal seeking of help (18) and affects the likelihood that they will seek help in the future (19). Unfortunately, many parents do not know the best way to approach NSSI in their children (20). Furthermore, NSSI has a series of serious consequences, not only for adolescents, such as permanent scars, rejection by peers, academic difficulties, and risk of suicide (21, 22), but also for parents and families (17). Compared with other parents, the parents of teenagers with NSSI have higher levels of stress and lower levels of satisfaction. Mothers of adolescents with NSSI also showed more symptoms of depression, anxiety, and stress (23). In addition, parent-child relationships change, posing challenges to the family unit (24). However, the impact of NSSI in adolescents on the family is often ignored (25). Therefore, it becomes particularly important to determine the understanding and the coping styles for NSSI of parents, as well as the impact of NSSI behaviors in teenagers on parents in China.

Currently, despite some progress being made on how the perceptions about NSSI of parents, most of the research on this topic has been performed in developed countries, and research exploring parents’ attitudes toward NSSI in adolescents and the seeking of help or support in China is been limited. The culture and the healthcare system in China are different from those in developed countries. The field of mental health in China started relatively late. The total amount of mental health resources has been at a low level for a long time, and human resources are scarce (26). Even two-thirds of counties did not have professional institutions for mental health services and the majority of people are short of mental health awareness (27). We intend to close these research gaps in this study. First, the study focuses on parents’ attitudes toward and perceptions of NSSI and its impact on families. We aim to generate information that could have a positive impact on parents and families and to explore the positive and negative significance of parents’ attitudes toward teenagers’ NSSI. Second, to understand some complex processes, qualitative research is the most appropriate method (28). Finally, the study aimed to provide the experiences of participants which could be used in the follow-up interventions to help other parents in the future.

## Methods

### Participants

Purposive sampling was used in the study. Parents of adolescents with NSSI were recruited from the child psychiatric ward of a tertiary hospital in China and were given informed consent forms. All the parents of teenagers meeting the inclusion criteria in the ward were invited. The inclusion criteria were as follows: 1) the participants had a biological child between 12 and 18 years old who were hospitalized for psychiatric disorders with a history of at least two NSSIs; 2) the children of participants do not have severe comorbidities and psychotic symptoms, such as hallucinations, delusion, etc.; 3) the participants were able to complete the interview with normal intelligence. Parents were excluded when they did not know whether the child had a history of NSSI and suffered from serious physical or mental illness and could not participate in the study. If parents were willing to participate in the study, they were given a month to call the researchers by telephone, and their children’s clinical data were retrieved. Whether adolescents exhibited NSSI behaviors, including cutting their wrists and thighs with knives and other sharp objects, poisoning themselves, or engaging in other behaviors causing bodily harm, was determined by the clinical data recorded by the psychiatrists during the first consultations. Parental mental state was based on the family history in their children’s clinical data.

Twenty-six parents were invited to participate in interviews. In total, 20 participants (16 mothers and 4 fathers) completed an interview. Six parents discontinued participation in the study, including three parents who suggested that we interview their children, two parents who refused to allow us to record, and one parent who was too emotional to continue the interview.

The study, conducted from August 2019 to December 2019, was approved by the medical ethics committee of the Second Xiangya Hospital of Central South University. All of the participants were informed of the purpose, methods, and privacy of the study and have signed informed consent forms.

### Procedures

An in-depth, semi-structured individual interview was conducted in the psychological interview room of the ward, and all of the participants’ children were inpatients. In the study, participants underwent an in-depth interview and a psychological consultation, and they received a crayon as a gift. The outline of the self-designed semi-structured interview was developed based on the literature (18, 24) and after discussions with psychiatrists and psychiatric nurses who had encountered adolescents with NSSI. Then, after consulting qualitative research experts, the outline of the interview was finalized. It covered 13 topics, some of which were as follows: 1) How do you know your child had an NSSI? 2) What was your first reaction after learning that your child had an NSSI? 3) What strategies did you use to cope with your child’s NSSI? 4) Have you persuaded your child not to hurt him/herself? 5) Are you afraid of outsiders learning about your child’s NSSI? Are you feeling shame? 6) Do you understand your child’s NSSI? 7) What do you think of your child’s hospitalization? and 8) Please describe how you usually get along with your children. Each interview typically lasted 40–50 min. All of the interviews were audio-recorded.

### Data Analysis

At the end of each interview, two researchers converted the audio recording into text and coded the data separately. One researcher has a background in psychology, and the other has a background in psychiatry. The general information was extracted in the form of a table. Data coding was performed in NVivo software, version 11. Thematic analysis was used and followed the six-step process by Braun and Clarke (29). Subthemes were formed by extracting the nodes repeatedly. After several discussions, the two researchers reached a consensus on the subthemes, which were agreed upon by all of the staff at the authors’ meeting.

## Results

Twenty parents were included in the study. **Table 1** displays the characteristics of the participants and their children. The mean age of the children was 14.5 years old (range: 12.0–18.0 years), and most were girls (85%). The average length of hospitalization was 5.6 d (range: 1–16 d) on the day of interview, including soon after the admission of adolescents (60%), soon after stabilization of symptoms (30%), and approaching the discharge (10%). Reasons for the hospitalization of adolescents were emotional disorders and repeated self-injury. Fifteen adolescents (75%) were diagnosed with nonpsychotic major depressive disorder (F32.2), three adolescents (15%) with nonpsychotic bipolar disorder (F31.4), and two adolescents (10%) with behavioral and emotional disorders with onset usually occurring in childhood and adolescence (F90–F98) according to the International Classification of Diseases 10th Revision (ICD-10). Fifty-five percent of the children with the duration of mental illness is less than one year, 30% of them have been diagnosed for 1–3 years, and 15% of them had more than 3 years. The time when they first engaged in NSSI was almost the same as the time when they received the diagnosis of mental illness. Self-injury methods included cutting (65%), head banging (10%), poisoning (10%), and a combination of methods (10%). Seventy percent of the children lived in towns or cities, and half were junior high school or high school students. It was found that 80% of the adolescents had one or more siblings, of which one teenager’s sister has a history of mental illness. Fifty percent of the parents’ education level were more than high school. The parenting style of parents are mentioned with parents’ authoritarian (40%) and the inter-generational parenting (25%).

**Table 1:** Participant characteristics.

**Age of child (years, SD, range)**	14.5 (2.0;12.0–18.0)
**Diagnosis of child**	
Depression	15 (75%)
Bipolar disorder	3 (15%))
Unspecified behavioral and emotional disorder	2 (10%)
**Gender of child**	
Female	17 (85%)
Male	3 (15%)
**Gender of interviewee**	
Female	16 (80%)
Male	4 (20%)
**Residence**	
City	14 (70%)
Countryside	6 (30%)
**Education of child**	
Middle school	10 (50%)
High school	10 (50%)
**Education of father**	
Less than high school	6 (30%)
High school	4 (20%)
More than high school	10 (50%)
**Education of mother**	
Less than high school	4 (20%)
High school	4 (20%)
More than high school	12 (60%)
**Marital status of participants**	
Married	14 (70%)
Divorced	5 (25%)
Widowed	1 (5%)
**The method of NSSI**	
Cutting	13 (10%)
Poisoning	2 (10%)
Head banging	2 (10%)
A combination of methods	2 (10%)

**Figure 1:**
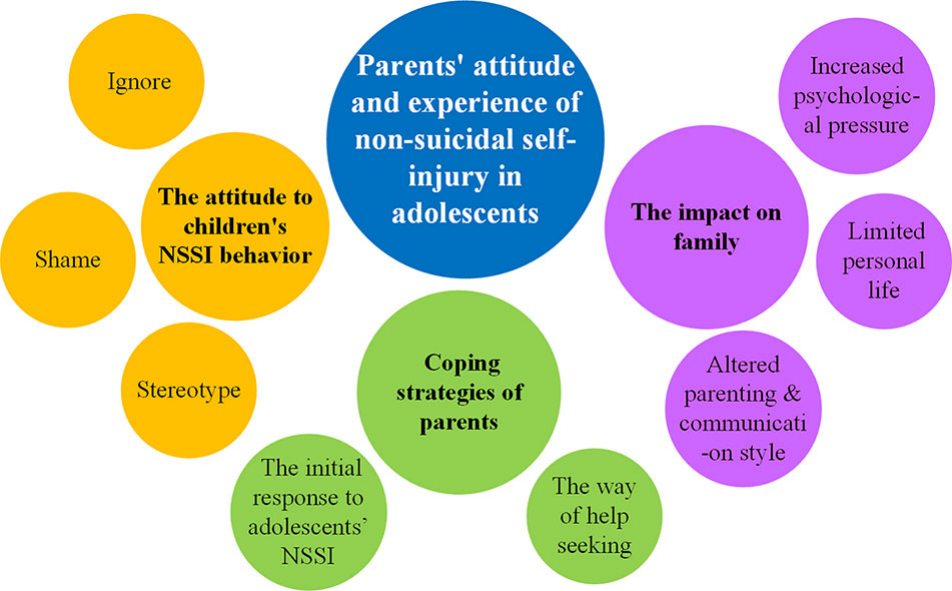
Thematic map of the three themes (middle circle) and eight sub-themes (small circle).

Through the thematic analysis, three themes and eight sub-themes were extracted: (1) the attitudes to children’s NSSI behaviors; (2) coping strategies of parents; and (3) the impacts on family (see **Figure 1**).

### Theme 1: The Attitudes to Children’s NSSI Behaviors

#### Subtheme 1: Ignorance

Many participants reported that NSSI is not very common, especially without a family history. For this reason, when they learned about their child’s NSSI, they ignored the behavior.

*“…Then she told me she wanted to see a psychiatrist, but I didn’t even think of it because this didn’t happen to any of the older kids in my family, so I didn’t care…” (P1)*

*“…When he told me in junior high school that he was a little mentally abnormal, we didn’t care. We asked him why, and he didn’t say. We still don’t know until now…” (P5)*

Other participants underestimated the severity of the NSSI and believed that their children would recover on their own.

*“…I know she is sick and very anxious. But I don’t think it’s a big deal…” (p4)*

#### Subtheme 2: Shame

Many parents did not know much about mood disorders and NSSI. Many thought that mood disorders were due to a problem with the brain and that their children were like “idiots”, the popular understanding being that such people exhibit inappropriate behaviors and language. Therefore, they felt too ashamed to tell others that they had a child with NSSI.

*“…After all, it is a mental illness, and others may have some bad opinions…” (p2)*

*“…Not many people know that my daughter is getting sick. I do not want to tell anyone. My family did not know before. I am just afraid that people will find out, and afraid that people will look down on my daughter…” (p1)*

Due to the stigma of mood disorders, some parents were reluctant to seek help from others, such as friends or colleagues. In addition, they worried that it would have a negative impact on their children’s further education, employment, and marriage.

*“…Because I was afraid that it would have an impact on her marriage, even if she recovered…”* *(p9)*

“…*I am afraid that being known by others will have an impact on the future…” (p3)*

#### Subtheme 3: Stereotype

Stereotypes about mood disorders caused misconceptions about them. Most of the parents thought mood disorders were not very serious and were not life threatening. In their opinion, teenagers do not experience any pressures, and they can go out and relax to cure a mood disorder because it is not a mental disease.

*“…When we took her on a trip, she was in a good mood and did not hurt herself, as if she had recovered from her illness…” (p2)*

*“…At the beginning, as soon as she said she was in a bad mood, I took her out to play and shop…” (p11)*

Some parents believed that if they did not pay too much attention to their children’s self-injury behaviors, their children would become better and their condition would not become more serious.

*“…Because I am afraid that if I pay too much attention to her self-injury, she will think she is more abnormal, so I dare not pay attention to her…” (p15)*

*“…I don’t think he’s a patient. I enlightened him. I never thought he was a patient…” (p3)*

A few parents often confused mood disorders with other mental illness and believed that only someone who is disoriented and has abnormal behaviors has a mental disorder and must go to the hospital for treatment.

*“…I think if you have this disease, you would not even know your parents and wander around the road. But she said everything clearly and knows everything, so I can’t believe that she is ill…” (p13)*

*“…Sometimes I don’t understand what she says to me. For example, she said before, ‘Mom, I seem to have depression.’ I said, ‘What the hell to depression, and depression is psychosis…’” (p16)*

Furthermore, when her granddaughter was in a slightly better mood or had no obvious signs of illness, one grandparent believed that taking drugs was harmful and would subjectively reduce the medication dose.

*“…Her grandmother thinks it must be bad to take too much medicine. One medicine was taken at night, and the dose was one and a half, and her grandmother threw away half of it…” (p10)*

### Theme 2: Coping Strategies of Parents

#### Subtheme 1: The Initial Response to Adolescents’ NSSI

When participants learned that their children had NSSI behaviors, they blamed their children because they were afraid that the child would commit suicide later and felt nervous.

*“…We blamed him because we were scared that he would commit suicide in the future too…” (p5)*

*“…At the beginning, I blamed her because I was a little nervous…” (p11)*

Some participants educated their children in their own way to persuade them not to hurt themselves again.

*“…I was worried and said that ‘Don’t hurt yourself’. I said,’ if you cut your hand again, I would cut it like you…’” (p16)*

*“We just talked to her a little more for fear that she would hurt herself again…” (p17)*

A few participants felt angry about their children’s NSSI behavior, because they believed that children hurt themselves in order to threaten their parents and achieve their own goals.

*“…She threatened me with cutting her wrists every time. What she wants me to do, if I don’t do it, she says she wants to cut her wrists. So I was angry and ignored her…” (p6)*

#### Subtheme 2: The Way of Help Seeking

Many participants did not know how to cope with the child’s NSSI behavior and did not take the child to a psychiatrist in a timely manner. They went to a professional institution for help only after their child or someone else requested it.

*“…Because none of my other kids had ever done this, I didn’t care. Later, she said she wanted to see a psychiatrist, so I took her to see a psychiatrist…” (p14)*

*“…At the beginning, I felt that she had a very stubborn character, and I just felt that her temper was getting worse, but we didn’t pay much attention to it. Later, her teacher also said that she had a bad temper and asked us to see a psychiatrist…” (p11)*

Some of the participants were more likely to take their children for a full-body examination when they find something unusual and the doctor suggested taking their children to a psychiatrist.

*“…She said she didn’t want to go to school, and said she was uncomfortable, so I took her for an inspection, but nothing was found…” (p1)*

*“…I took her to the largest hospital in my hometown again. The doctor said that if she is always in such situations, it must be a psychological problem…” (p13)*

### Theme 3: The Impacts on Family

#### Subtheme 1: Altered Parenting and Communication Styles

Most of the participants said that they were less likely to lose their temper and were more able to control their emotions. Therefore, they no longer had face-to-face confrontations with their children.

*“…I am more able to control my emotions, and I will not show that I am annoyed with her…” (p15)*

*“…Actually, I have a temper, but I didn’t let it out. It’s better talk to her in a nice way…” (p20)*

Some participants reported being more patient with and paying more attention to their children. Furthermore, they were willing to attempt to communicate with their children as friends rather than as authority figures, like they used to.

*“…When I speak to her now, my voice is a little lower and softer. I will deliberately pay attention to the content and my way of talking to her…” (p2)*

*“…I’m a little more patient than before, and most of the time I don’t blame her like I used to…” (p11)*

A few participants mentioned that they would develop together with their children and improve themselves. They did not have high expectations for the future and would not pressure their children.

*“…I think we should grow up together with our children, he should learn the method of catharsis, and we parents should also learn to change the way of education, the way of communication…” (p5)*

*“…I just hope she can support herself in the future. I have regarded her as a friend rather than a child…” (p6)*

#### Subtheme 2: Limited Personal Lives

Many participants said that, since they had learned that their child was ill, they had asked for leave or had quit their jobs to stay with their children all the time. A few participants changed jobs, accepting less pay so that they could stay with their children.

*“…I haven’t managed the business in my shop since she was ill…” (p4)*

*“…I asked for leave from work to accompany her/him…” (p7, 14, 18)*

*“…I worked in the hotel next to his school, and I got a job in the housekeeping department because I’m afraid he won’t eat and take medicine on time…” (p3)*

Other participants mentioned that they spent less time chatting and partying with their friends and spent more time with their children.

*“…They spend less time chatting and socializing with their friends and spend more time with their children…” (p2)*

#### Subtheme 3: Increased Psychological Pressure

Most of the participants had high medical expenses, and some of the participants had quit their jobs. Furthermore, most of the children were in the middle and high school, which is a prime period of learning, but the illness can cause academic performance to suffer.

*“…I’m a little depressed. The child has been excellent since he was a child; suddenly, he said that he would never go to school again. I think it’s a pity, so I’m a little depressed…” (p12)*

*“…In addition to hospitalization and medication costs, sometimes she wants to go out to relax and go out for fun, so the financial pressure is very great…” (p19)*

Other participants mentioned that they sleep less and that their sleep quality has worsened since learning about their child’s NSSI. A few participants felt guilty about their child’s behavior. Several of the participants reported that they even felt broken and depressed.

*“…It’s the biggest blow to me. I feel like I’m a failure and I think it’s my own problem. I feel devastated…” (p7*)

*“…I didn’t go to bed until yesterday. I didn’t sleep much the previous few days. I was afraid she would hurt herself.” (p20)*

*“…I’m very depressed. But I was very optimistic and positive in front of her…” (p2)*

A few participants felt that the future was a little bleak because the effect of the treatment was not obvious. In addition, they worried about the recurrence of their child’s illness.

*“…I am afraid that she will have a relapse in the future because the recurrence rate is so high…” (p8)*

## Discussion

To our knowledge, the study is the first to investigate parents’ attitudes toward and perceptions of adolescents with NSSI behaviors in China. Our research found that parents do not know how to cope with their children’s NSSI behaviors and lack relevant knowledge about NSSI in adolescents. Second, the initial response of parents is often not to take their children to the hospital; this option is, in fact, sometimes the last option for parents. In addition, a child’s NSSI has some impacts on the parents, altering their parenting, limiting their personal lives and creating a parental burden.

The results showed that most parents lack knowledge about NSSI in adolescents. On the one hand, some of them had never heard of NSSI behaviors before their children engaged in NSSI and underestimate its severity; thus, they ignored it and believed that things will improve spontaneously. This attitude is consistent with Oldershaw and colleagues’ study (18). On the other hand, some participants regarded their children’s NSSI behavior as shameful, and they were therefore reluctant to mention their child’s NSSI history to others. This may be one of the hidden factors influencing parents’ lack of knowledge of NSSI. Moreover, many participants have stereotyped conceptions about mental illness. They believe that those suffering from mental illness are “lunatics” and have abnormal speech and behaviors. Therefore, it is difficult for them to equate NSSI with mental illness.

Moreover, in agreement with McDonald’s study (20), most of the parents do not know the best coping strategies for children’s self-injury behaviors. At the beginning, they tended to confuse self-harm with adolescent rebellion, so they blame and educate to persuade children not to self-harm again. Parents did not address the issue until the child or the teacher or the doctor suggested that the child should go to a psychiatrist for treatment. In addition, we found that there are many barriers to help-seeking for adolescents with NSSI. Some parents fear receiving negative reactions and disrupting academic achievement, employment, and marriage (19, 30), or they simply misunderstand NSSI. As a result, when children mention going to the hospital for treatment, parents’ perceptions conflict with their children’s perceptions. In this study, we found that most teenagers with NSSI wounds take the initiative to ask for help, which stands in contrast to the findings of Pumpa and colleagues (31), perhaps because our participants were all parents of hospitalized patients.

After learning about NSSI in adolescents, the parents underwent changes. In terms of parenting strategy, they altered their parenting and communication styles due to fear of the child’s repeated self-injury. Consistent with Byrne’s research (32), some parents reported being more patient and gentle with their children and avoiding conflicts with them. Nevertheless, parents mentioned that communicating with their children, especially about NSSI, was a difficult process. In addition, we found that parents are under great parenting stress. On the one hand, they face a great financial burden. In addition to the expenses of medication, hospitalization, psychological counseling, etc., in order to make their children happy and stop hurting themselves, parents address their children’s various desire for shopping, traveling, etc. On the other hand, parents felt increased psychological pressure due to fear that their child would have a relapse and neglect their studies as well as guilt and depression about their child’s NSSI.

Regarding existing problems, some interventions have been developed for NSSI behaviors in adolescents consisting of pharmacotherapy and psychotherapies, such as cognitive behavior therapy, interpersonal therapy, and family therapy, etc (33, 34). These interventions have made some progress in reducing the incidence and frequency of NSSI to some extent. However, these studies also have some limitations, such as low participation rates, a lack of a specific focus on NSSI, small sample sizes, and few high-quality randomized controlled trials. According to our findings, considering the characteristics of adolescents with NSSI and their parents, the interventions with low-threshold access, low stigma, and high confidentiality are needed. Online interventions might be more acceptable than in-person interventions. Second, the parents of adolescents with NSSI are under great parenting pressure, and their lives are impacted by their children’s conditions. Intervening parents and children together would be more effective in helping families with adolescents who engaged in NSSI.

Currently, in mainland China, the treatments for inpatients who engaged in NSSI are not optimistic. For one thing, the mental health system in China is understaffed. As of the beginning of 2015, the number of psychiatric beds was approximately 1.71/100,000 population, which was lower than the global average of 4.36/100,000 (27). Psychiatrists and psychiatric nurses are the main professionals providing mental health services, with few clinical psychologists, psychotherapists and social workers involved in China. The resources of mental health services are mostly distributed in cities, with few in rural areas. Moreover, the interventions in China mainly are in the research stage, and there is a lack of intervention programs specially aimed at self-injury (26). As for the treatment methods, pharmacotherapy is the domain approach, supplemented by psychotherapy. Generally, it mainly treats the mental illness first which co-occurs with NSSI, such as depressive disorders or bipolar disorder. Furthermore, there is no national intervention plan addressing suicide and NSSI in children and adolescents.

## Limitation

In the study, our findings could only be generalized to a certain extent because the participants are all the parents of inpatients. Their attitudes and perceptions might more reflected those of parents whose children have been referred to medical institutions. In addition, most of our participants were mothers because most teenagers were accompanied by their mothers in hospital. This is influenced by the culture, that is, women are the main providers of informal care for family members (35). Therefore, our results might be more representative of mothers’ attitudes. Future studies may focus on fathers’ parenting, attitudes, and perceptions. Second, we did not go through a rigorous mental health evaluation of parents and did not use validated instruments to measure parenting stress. In future research, quantitative explorations could be undertaken to understand the parenting stress and mental health status of parents. Third, the psychological factors and temperament of the adolescents were not involved. It is suggested to explore the influence of psychological factors and temperament of adolescents on NSSI and parents in the follow-up studies.

## Conclusions

We investigated parents’ attitudes toward NSSI in Chinese adolescents and the impacts on their parents in mainland China. The results showed that parents lack the knowledge about NSSI and its treatment and are suffering from great emotional stress. According to these findings, we recommend expanding the popularization of knowledge to increase awareness of NSSI in adolescents and help them to seek professional support in a timely manner. In addition, the well-being of parents of adolescents who have engaged in NSSI require attention, indicating that much research is still needed to improve the well-being of both parents and families. The study supported that online interventions adapted to China’s sociocultural climate could be a viable option.

In future research, it would be meaningful to expand the range of participants to family members or the adolescents themselves to understand more perspectives and the impact of NSSI on additional groups. If a community sample could be recruited, a more comprehensive understanding of the attitudes of Chinese parents toward NSSI in adolescents could be obtained. In addition, long-term follow-up studies are necessary to understand the long-term effects of NSSI behaviors in adolescents on families or changes to parent-adolescent relationships.

## Data Availability Statement

The raw data supporting the conclusions of this article will be made available by the authors, without undue reservation, to any qualified researcher.

## Ethics Statement

The study, conducted from August 2019 to December 2019, has been approved by the Medical Ethics Committee of the Second Xiangya Hospital of Central South University. All participants have been informed of the purpose, methods, and privacy of the study and have signed informed consent forms.

## Author Contributions

JO, YL, and RC designed the study. Data curation: XF, JL, and YP. Formal analysis: XF and JY. Writing—original draft: XF. Writing—review and revising: XL, YS, JO, and YL. XF, JO and YL obtained the funding. All authors contributed to the article and approved the submitted version.

## Funding

The study is funded by the National Natural Science Foundation of China (No. 81974217 and 81873806) and Fundamental Research Funds for the Central Universities of Central South University (No. 2020zzts840).

## Conflict of Interest

The authors declare that the research was conducted in the absence of any commercial or financial relationship that could be construed as a potential conflict of interest.
