# Supplementary material for: Parents’ Attitudes Toward and Experience of Non-Suicidal Self-Injury in Adolescents: A Qualitative Study
Source: Front Psychiatry. 2020 Jul 15;11:651. doi: 10.3389/fpsyt.2020.00651 (PMC7373789; doi:10.3389/fpsyt.2020.00651)
Supplement: Supplementary file 1 [file Table_1.docx]

Supplementary Material

# Supplementary Tables

# Table 1 General demographic of participants

| Participant | | | | | | | | Child of participant | | | | |  |  |
| --- | --- | --- | --- | --- | --- | --- | --- | --- | --- | --- | --- | --- | --- | --- |
| Participant | Gender | Nationality | Residence | Education | Education of spouse | Marital status | Number of Children | Age | Gender | Education | Diagnosis | The way to NSSI | The length of hospitalisation during the interview | The length of hospitalisation |
| 1 | female | Han | Town | Primary school | Middle school | Married | 4 | 16 | female | High school | Depressive disorder | Cutting | 10 | 14 |
| 2 | female | Han | City | High school | High school | Married | 2 | 17 | female | High school | Depressive disorder | Cutting | 8 | 19 |
| 3 | female | Han | Countryside | Primary school | Primary school | Married | 2 | 16 | female | High school | bipolar disorder | Refusing to eat | 5 | 22 |
| 4 | female | Han | Town | High school | High school | Married | 1 | 12 | female | Middle school | Depressive disorder | Cutting/Pinching | 6 | 26 |
| 5 | female | Han | City | High school | High school | Divorce | 2 | 17 | female | High school | bipolar disorder | Cutting | 5 | 10 |
| 6 | female | Han | Countryside | Primary school | High school | Divorce | 2 | 18 | female | High school | Depressive disorder | Cutting | 16 | 18 |
| 7 | female | Han | Countryside | Middle school | Middle school | Divorce | 2 | 16 | female | High school | Unspecified behavioural and emotional disorder | Poisoning | 2 | 13 |
| 8 | female | Han | Countryside | Middle school | Middle school | Married | 2 | 14 | female | Middle school | Depressive disorder | Cutting | 2 | 8 |
| 9 | female | Han | Town | Middle school | Middle school | Married | 2 | 17 | female | High school | Unspecified behavioural and emotional disorder | Punching the wall | 1 | 8 |
| 10 | female | Han | Countryside | Primary school | Primary school | Married | 2 | 15 | female | Middle school | Depressive disorder | Cutting | 10 | 18 |
| 11 | female | Han | City | College | College | Divorce | 1 | 12 | female | Middle school | Depressive disorder | Cutting | 10 | 19 |
| 12 | female | Han | Town | High school | High school | Married | 2 | 15 | female | High school | Depressive disorder | Cutting | 3 | 8 |
| 13 | female | Han | Countryside | Primary school | Primary school | Married | 4 | 12 | female | Middle school | bipolar disorder | Cutting | 4 | 35 |
| 14 | female | Han | Town | Middle school | High school | Married | 2 | 13 | female | High school | Depressive disorder | Cutting | 4 | 27 |
| 15 | female | Han | City | Middle school | Middle school | Married | 2 | 12 | female | Middle school | Depressive disorder | Cutting | 3 | 18 |
| 16 | female | Han | Town | Middle school | College | Bereavement | 1 | 15 | female | High school | Depressive disorder | Cutting | 1 | 18 |
| 17 | female | Han | City | College | College | Divorce | 1 | 12 | female | Middle school | Depressive disorder | Cutting | 2 | 14 |
| 18 | female | Han | Town | Primary school | Primary school | Married | 2 | 13 | female | Middle school | Depressive disorder | Head banging | 8 | 18 |
| 19 | female | Han | City | College | College | Married | 3 | 15 | female | Middle school | Depressive disorder | Head banging | 6 | 14 |
| 20 | female | Han | Town | Primary school | Primary school | Married | 2 | 13 | female | Middle school | Depressive disorder | Cutting/Poisoning | 5 | 19 |
